# Quantum technologies in particle physics

**DOI:** 10.1098/rsta.2021.0072

**Published:** 2022-02-07

**Authors:** Steven D. Bass, Erez Zohar

**Affiliations:** ^1^ Kitzbühel Centre for Physics, Kitzbühel Austria; ^2^ Marian Smoluchowski Institute of Physics and Institute for Theoretical Physics, Jagiellonian University, Kraków, Poland; ^3^ Racah Institute of Physics, The Hebrew University of Jerusalem, Givat Ram, Jerusalem, Israel

This theme issue concerns exciting new developments at the interface of quantum and particle physics, together with quantum technologies and their application to fundamental particle physics. This is a new priority area for European and US research as identified by the United States DoE and European funding authorities as well as by CERN. The theme issue brings together key people involved in this effort with a positive outlook to the future. The volume explores fresh synergies between quantum and particle science and the potential for exciting new cross disciplinary collaboration.

Particle physics today is characterized by the success of the Standard Model [1], including the recent discovery of the Higgs boson at the Large Hadron Collider, LHC, at CERN. So far there is no evidence for extra particles or interactions in the energy range of present experiments. However, some new physics is required to explain the tiny neutrino masses, baryogenesis with extra CP violation, and the phenomena of dark matter and dark energy. The energy scale of this new physics is presently unknown, with the quest for this new physics a prime topic for both experimentalists and theoreticians.

Theoretically, non-abelian gauge theories require non-perturbative treatment because of strong coupling and confinement dynamics in the infrared. Inspired by Feynman’s initial intuition of quantum computing [2], task-specific quantum simulators are being used in a first step to simulate a quantum system using another more controllable one, with recent vision outlined in [3]. Quantum simulators are being developed to study lattice gauge theories as a new tool to investigate non-perturbative dynamics at strong coupling. These may eventually lead to new insight into difficult problems like quark confinement and the properties of dense nuclear matter, e.g. neutron stars. The latter cannot in general be investigated using conventional lattice Monte Carlo techniques because of sign problem issues.

The push to higher energies and luminosities in collider experiments comes with huge datasets requiring considerable improvements in computing power. Quantum machine learning- and quantum computing-based ideas are being explored in connection with these challenges. In parallel, the next generation of gravitational wave experiments may be sensitive to particle physics phenomena in the early Universe as well as to neutron star and black hole dynamics. Quantum ideas enter here with cold atom technologies being considered as an experimental tool in gravitational wave detection. On the theoretical side, there are ideas that black holes may behave like quantum computers in how they process information.

To the deepest level probed by experiments, Nature is described by quantum physics together with gauge symmetries and Poincaré invariance. Where do these come from? Gauge symmetries and Lorentz symmetry with limiting velocities are known to be emergent in the low energy limit of quantum many-body systems characterized by long-range entanglement and topological order. Might the gauge symmetries of particle physics also be emergent, with gauge bosons, leptons and quarks ‘dissolving’ deep in the ultraviolet, close to the Planck scale? Considering this scenario opens possible new synergies between particle physics and ideas in quantum information and condensed matter physics, waiting to be fully explored. It is plausible that quantum physics including particles with Fermi–Dirac statistics may, at a deeper level, itself be emergent from some more primordial physics.

The articles in this volume fall into four different categories spanning thinking and activity in these topics. Fundamental issues on the origin of quantum phenomena and gauge symmetries are discussed by Wetterich, Dvali and Bass. Ideas about quantum entanglement in high-energy QCD collisions are considered by Aidala and Rogers and by Kharzeev. Quantum simulations of quantum field theories and particle physics are covered in a series of theoretical papers by Lewenstein and collaborators, Zohar, Wiese, Jansen and collaborators, and Montangero and collaborators. Finally, in direct contact with experiments, Gray discusses the status of new ideas on the application of quantum computing techniques to LHC collider experiments. Buchmueller, Ellis and collaborators discuss a new programme to use cold atom technology in next-generation gravitational wave detection experiments.

Wetterich considers the possible origin of fermions and quantum phenomena from classical bits using a probabilistic cellular automaton model working in 1+1 dimensions [4]. Quantum mechanics can be understood as a particular case of classical statistics. Given the quantum world, there are the important issues of the capacity of quantum information storage and its retrieval time in gauge field theories. This is discussed by Dvali with information capacity quantified by the microstate entropy [5]. Universal bounds are derived for quantum gauged systems and for black holes which are shown to operate within the same rules of maximal information storage capacity. Where do gauge theories of particle physics come from? Bass discusses the Standard Model as a possible emergent system exploring parallels with phenomena in condensed matter physics, also with application to Higgs systems and the cosmological constant or vacuum energy puzzle [6]. The cosmological constant may enter as a higher dimensional term in the action associated with emergent symmetries and the symmetry of the metric. Emergent gauge systems in condensed matter systems are known since the pioneering work of Anderson and collaborators with the low energy limit of the Fermi–Hubbard model at half filling [7].

Quantum entanglement in QCD high-energy collisions is discussed in two articles by Aidala and Rogers and by Kharzeev. In the first contribution, Aidala & Rogers consider QCD factorization and its breaking in terms of decoherence and entanglement [8]. Entanglement is exhibited by the Collins effect in QCD spin physics where quark transverse momentum becomes important. Next, Kharzeev relates the entanglement entropy in high-energy QCD processes to the Fock state probability distribution [9]. At large rapidity, the hadron state becomes maximally entangled. The probabilistic parton distribution breaks down for spin processes where the phases of Fock state components are controlled. Kharzeev suggests that entanglement entropy may be emergent on the light cone as a general phenomenon that should exist even when the parton model does not apply, e.g. at strong coupling.

Quantum simulations of gauge theories have been pioneered using a number of techniques in 1+1 dimensions, e.g. the bosonic and fermionic Schwinger models, and studying properties such as entanglement and confinement. Lewenstein and collaborators focus on the interface of cold atom technologies and quantum simulations [10]. Promising results suggest that simulations of non-abelian gauge theories in this approach will soon be possible, including with experimentally friendly quantum simulators, to look at models relevant to both condensed matter and particle physics. Next, Zohar discusses the theoretical challenges of extending both analogue- and analogue–digital-type quantum simulations from 1+1 to higher dimensions [11], e.g. relevant to particle physics experimental phenomena. Wiese discusses quantum link models and D-theory as a promising resource efficient framework for the quantum simulation and computation of gauge theories [12]. Here gauge fields are represented by discrete quantum variables which reside in a finite-dimensional Hilbert space. With D-theory, the continuum gauge theories emerge by dimensional reduction from the collective dynamics of discrete quantum variables. For quantum computing, control of errors with quantum algorithms is an important issue. Jansen and collaborators discuss two recent algorithmic advances for simulating particle physics phenomena using intermediate scale quantum devices [13]. Tensor network methods have been extensively used for studying lattice gauge theories in recent years, allowing for computations beyond the capabilities of Monte Carlo methods. These are discussed here by Montangero and collaborators with prime focus on the particular approach of tree tensor networks [14].

With direct application to LHC experiments, Gray discusses the challenges of data pile up and the computing power needed for the high luminosity LHC upgrade at CERN, HL-LHC and beyond [15]. First experience with quantum computers looks promising. These may develop to providing a solution to the challenges of processing the huge datasets expected from future experimental runs.

Finally, Buchmueller, Ellis and collaborators consider new ideas how cold atom interferometry techniques might play a vital role in future next-generation gravitational wave detection experiments [16]. These experiments might reveal essential new information on, e.g. the origin of black holes in the early Universe, phase transitions in the very early Universe and helping in the search for ultra-light dark matter candidates. Initially planned to be Earth based, atom interferometer gravitational wave detectors might later be launched into space, the AEDGE project being discussed in connection with ESA.

We hope that bringing these topics together in the same volume will open new synergies between quantum and particle physics, and inspire a new generation of excellent young scientists to join.

The idea for the present volume was conceived during discussions that arose at the Humboldt Kolleg meeting *Discoveries and Open Puzzles in Particle Physics and Gravitation*, held in Kitzbühel Austria in June 2019.

## Data accessibility

This article has no additional data.
